# Resilience in drug recovery: insights from treatment and rehabilitation programmes in Malaysia

**DOI:** 10.1186/s13011-026-00745-2

**Published:** 2026-07-15

**Authors:** Siti Zulaikha Mustapha, Yarina Ahmad, Nur Amalina Aziz, Amanda Balqis Mohd Mazalan, Nor Azira Ayob, Nor Farizah Johari

**Affiliations:** 1 Faculty of Administrative Science and Policy Studies, Universiti Teknologi MARA, Machang, Kelantan, 18500 Malaysia; 2 Institute of Leadership and Development, Universiti Teknologi MARA, Lebuh Enstek, Bandar Enstek, Nilai, Negeri Sembilan, 71760 Malaysia; 3 Faculty of Administrative Science and Policy Studies, Universiti Teknologi MARA, Shah Alam, Selangor 40450 Malaysia; 4 Faculty of Business and Management, Universiti Teknologi MARA, Segamat Campus, Segamat, Johor 85000 Malaysia; 5 Faculty of Administrative Science and Policy Studies, Universiti Teknologi MARA, Seremban 3, Seremban, Negeri Sembilan 70300 Malaysia

**Keywords:** Drug, Treatment and Rehabilitation, Resilience, Cure and Care Clinics, Effectiveness

## Abstract

**Background:**

This paper explores key factors influencing individuals’ resilience upon experiencing drug use disorder and the barriers to resilience that they face, based on insights from drug treatment and rehabilitation programmes implemented in Malaysia. Despite continuous efforts, drug use disorder remains a persistent issue linked to broader social and security concerns.

**Method:**

A qualitative case study approach was employed, involving four Cure and Care 1Malaysia (C&C1M) clinics across Peninsular Malaysia. A total of 37 participants, including both clients and service providers, were involved in this study. Data were analysed using thematic analysis with the support of NVivo software.

**Findings:**

The findings indicate that participants’ resilience is shaped through treatment and rehabilitation process. Two key factors were identified: (1) the guidance, support, and trust that clients receive during recovery; and (2) the need for post-treatment programme enhancement. Whereas, the barriers to resilience consist of (1) individuals’ self-reliance and (2) limited institutional support. Therefore, this paper argues that it is important to enhance the resilience of individuals who have the experience of being involved in drug use disorder. This can be done by: (1) emphasising aftercare services; (2) empowering clients; and (3) strengthening policy implementation, monitoring, and evaluation. The findings offer important implications for improving drug treatment policies and strengthening long-term recovery strategies.

**Conclusion:**

This study highlights the importance of strengthening resilience as part of recovery efforts. Sustained recovery requires continuous support beyond institutional settings, through the involvement of multiple stakeholders.

**Supplementary Information:**

The online version contains supplementary material available at https://doi.org/10.1186/s13011-026-00745-2.

## Background

Drug use disorder remains a global issue affecting many countries. Its impact continues across generations, with evolving patterns of drug abuse, trafficking, and changing characteristics of individuals involved, such as age, gender, education level, and employment status. Although various strategies have been implemented, drug treatment and rehabilitation programmes remain among the primary approaches to addressing use disorder, abuse, and dependency.

Drug-related issues are often interconnected with organised crimes and broader social challenges, posing significant threats to national safety and security [1, 2]. Malaysia is not exempted from this scenario. Continuous efforts have been undertaken at both national and global levels to address drug-related issues. For instance, the Sustainable Development Goals (SDGs), particularly indicator 3.5.1, emphasise the importance of treatment coverage for substance use disorders, coordinated by the World Health Organization and the United Nations Office on Drugs and Crime [3]. Despite these efforts, addressing drug use disorder requires a holistic and sustained approach that integrates prevention, enforcement, socio-economic considerations, and rehabilitation strategies.

### The Malaysian drug treatment and rehabilitation programmes

In Malaysia, drug is recognized as the number one enemy, and it has been a long-standing issue that is becoming more complex and intertwined with many other problems and crimes. Based on drug addiction statistics from 2018 to 2023, the number of individual who involved with drug use disorders such as addiction had increased by 5.3%, from 130,788 cases in 2018 to 145,526 cases in 2023 [4]. This statistic shows that the number of individuals involved in drug use disorder in Malaysia are a never-ending problem, regardless of the various efforts undertaken by the government, private sector, non-governmental organisations, and others.

Historically, in the 4th Malaysia Plan (1981–1985), the Malaysian government began to focus on drug treatment and rehabilitation, establishing Persatuan Salahguna Dadah (PEMADAM) and drug treatment and rehabilitation centres under the Welfare Department Service [5]. In the 5th Malaysia Plan (1986–1990), the government introduced drug treatment and rehabilitation centres as part of the home affairs’ security efforts. As a result, security sectors allocated the expenditure for these centres, and the 5-year Anti-drug Action Plan (1985–1989) framework was established [6].

Then, in the 8th Malaysia Plan (2001–2005), the government started to embrace new approaches to dealing with drugs and enhanced community empowerment [7]. Subsequently in the 9th Malaysia Plan (2006–2010), the government launched the Transformation Programme and established the Special Cabinet Committee on Eradication of Drugs to coordinate and implement drug eradication efforts [8]. The cabinet later approved a drug treatment bill in the 12th Malaysia Plan (2021–2025), empowering the National Anti-Drugs Agency to authorise officers to apprehend, treat, and rehabilitate “drug dependents or misusers.” This amendment was to improve the recovery of individuals from drug-related health problems [9]. However, the effectiveness of drug treatment and rehabilitation programmes is highly subjective: no single approach or effort can cure drug use disorder. The government’s continuous efforts and proactive intervention aim to ensure the eradication of drug abuse and drug use disorder, particularly in Malaysia.

### Drug treatment and rehabilitation: cure & care 1 Malaysia clinics

In 2010, the Malaysian government implemented the Cure and Care Model as part of its transformation project, specifically targeting the reduction of crimes as one of the National Key Result Areas (NKRA). The overhaul of the treatment and rehabilitation programmes hads significantly improved the delivery of services, particularly to the intended recipients. The model was divided into five distinct entities: the Cure & Care 1Malaysia Clinic (CC1M), Cure and Care Service Centre (CCSC), Cure and Care Rehabilitation Centre (CCRC), Cure and Care Vocational Centre (CCVC), and Caring Community House (CCH). Cure and Care was a holistic strategy that encompassed all the fundamental elements of service settings provided by NADA such as inpatients, outpatients, clinical treatments, methadone substitution therapy as well as provision of referral and advocacy. The objective was to restore patients to a productive, functional, and healthy state [10].

The Cure & Care 1Malaysia Clinics were specifically designed to enhance client experience by reducing waiting time, shortening treatment length, and providing a wider range of medication-assisted detoxification options [11]. After six years since its inception, the treatment and programmes had shown promising results in terms of the number of clients served. The National Anti-Drugs Agency [12] reported that the number of clients enrolled in the treatment and rehabilitation programmes climbed from 5,864 in 2010 to 14,436 in 2013. In particular, the number of clients for outpatient services increased to 4,138. On April 12th, 2021, the centres were renamed to the Narcotics Rehabilitation Clinics (PUSPEN, Pusat Pemulihan Penagihan Narkotik). The treatment and rehabilitation methods of the service have also been rebranded and changed to align with the evolving needs of patients, as well as the direction set by the leadership and other pertinent aspects.

### Clients’ resilience in combating drug use disorder

Since the year 2000, scholars from numerous fields have conducted studies on resilience [13–16]. These scholars believe that resilience is a defence mechanism that can help individuals prevent themselves from relapsing and maintain their recovery. Resilience is a dynamic process that can prevent individuals from engaging in drug-related activities. The recovery process for individuals struggling with drug use disorder is primarily dependent on they themselves while other factors such as social and economic factors play a secondary role [13]. The characteristics of resilient individuals are those with positive emotions, realistic optimism, and active coping mechanism with stress, high coping self-efficacy, self-esteem, empathy, kindness, and compassion, apart from having the ability to change negative thoughts into positive ones and secure attachment, practicing a healthy lifestyle, and being able to survive in the community [13, 17, 18].

Resilience is also one of the main factors that can increase the effectiveness of preventing drug abuse, drug relapse, and drug triggers [19]. Thus, most scholars suggest that resilience should be developed in drug treatment and rehabilitation programmes, drug enforcement, and prevention approaches, as individuals’ resilience regardless of their gender, age, education background, health status, and other criteria is a powerful factor against any drug use-related disorder [20, 21]. Resilient individuals are also proven to have a lower relapse risk which will ensure their continuous recovery from time to time [17].

Drug treatment and rehabilitation programmes are widely recognised as effective mechanisms for addressing drug use disorders and facilitating the reintegration of individuals into their familial, occupational, and community environments [22]. While these programmes demonstrate success in supporting initial recovery and social reintegration, their effectiveness in ensuring sustained recovery remains a critical concern. In this regard, resilience emerges as a crucial yet underexplored dimension in strengthening long-term recovery outcomes. Although resilience has been examined across various contexts, there remains limited empirical integration of resilience within the evaluation of drug treatment and rehabilitation effectiveness. This study addresses this gap by focusing on how resilience can be developed and embedded within rehabilitation processes to enhance sustained recovery. Specifically, this paper seeks to uncover key resilience factors that strengthen the capacity of individuals with drug-related problems including abuse, use disorder, and dependency to maintain recovery and reduce the risk of relapse.

## Method

### Study design

A qualitative study using a case study approach was adopted in this study, focusing on four Cure & Care 1Malaysia Clinics representing Peninsular Malaysia. A case study is indicated to be a useful approach to a thorough exploration of drug treatment and rehabilitation services received and provided by the participants (both clients and service providers) in the centres [23]. Through the qualitative approach, the participants were made to understand the researchers’ ethical and formal procedures upon gathering views and feedback on their experience while dealing with the drug treatment and rehabilitation programmes.

### Study setting

During the implementation of Cure and Care Model from 2010 until 2021, there had been twelve (12) C&C1M Clinics throughout Malaysia. The study, nevertheless, only involved four C&C1M Clinics, located in Peninsular Malaysia. The C&C1M clinics namely Sungai Besi, Bukit Mertajam, Tampin, and Kota Bharu were selected to represent the cases in the central, south, east and west zone to ensure the rigour findings of the study.

### Clinics’ profiles

Established in 2010, the Cure and Care 1Malaysia Clinics served as the drug treatment and rehabilitation centres in Malaysia. However, on April 12th, 2021, the centres waere renamed the Narcotics Rehabilitation Centres or PUSPEN (Pusat Pemulihan Penagihan Narkotik). The Cure and Care 1Malaysia Clinics provided comprehensive drug treatment and rehabilitation services comprising three main components namely psychosocial interventions, medical treatment, and vocational training. Psychosocial interventions included relapse prevention, early recovery programmes, familial and spiritual support, counselling, and peer support activities. Medical treatment encompassed detoxification, methadone maintenance therapy, psychiatric care, treatment for infectious diseases, and other healthcare services. In addition, clients were offered vocational training, entrepreneurship programmes, and job placement support to facilitate their reintegration into society. The profile of each clinic is as follows:

#### Clinic 1

The C&C1M Clinic Sungai Besi was the first drug treatment and rehabilitation facility to use the Cure & Care Model, commencing in 2010. Previously known as Persada Sungai Besi, C&C1M Sungai Besi later changed its name to PUSPEN Sungai Besi. The primary objectives of this centre are to offer drug treatment and rehabilitation programmes to clients and targeted groups, as well as to give consultation services to the public regarding drug treatment and rehabilitation matters.

#### Clinic 2

The C&C1M Clinic Kota Bharu is a significant stakeholder in the Cure & Care project in 2010. The facility is situated in Pengkalan Chepa, Kota Bharu. Treatment and rehabilitation can be divided into two categories: inpatient, where individuals stay at the centre for a specific duration, and outpatient, where individuals attend appointments on a scheduled basis.

#### Clinic 3

The C&C1M Clinic Bukit Mertajam is another facility that was established in 2010. Formerly, this centre was referred to as PUSPEN or Narcotics Treatment Centre. Situated in Bukit Mertajam, Penang, this facility offers drug treatments and rehabilitation programmes to a diverse group of clients, including Malays, Chinese, and Indians.

#### Clinic 4

The C&C1M Clinic Tampin is situated in the southern region of Malaysia, and the majority of its clients consist of individuals who are either admitted to the centre or receive treatment as outpatients. These clients primarily come from the southern states of Johor and Kuala Lumpur. This centre exhibits a considerable degree of similarity to the other three centres that were part of this study. This treatment and rehabilitation module for individuals with drug use disorder offered at the centre emphasizes vocational skills. This is due to the wide and large area of the centre and the fact that this centre is geographically suitable for plantation activities.

### Study participants, criteria, and sampling

This case study involved a total of 37 participants, including both the receivers and providers of the services. The receivers refer to the clients or patients, who were the individuals involved in drug use disorder. The participants involved had experienced drug use disorder and relapses, whether they were reported or not by the centres. There was no age limitation or restriction imposed on the respondents of this study. The participants included adolescents (13–18 years old), youths (19–39 years old), and adults (40 years old and above). The participants selected had also received any types of drug treatment and rehabilitation programmes at the C&C1M Clinics, either as inpatients and outpatients.

Whereas, the providers included the C&C1M Clinic staff and representatives from the clinics, the National Anti-Drugs Agency (NADA), and the Ministry of Home Affairs, Malaysia (MOHA). The selected providers were the responsible parties dealing with drug-related issues in Malaysia. The participants also had the experience of dealing with the implementation of drug treatment and rehabilitation programmes at the C&C1M Clinics. They had at least one (1) year of work experience in the job scope. No age or gender requirement was imposed on the participants (refer to Table 1).

**Table 1:** Criteria of the participants

Item	Criteria of the participants	
	Client/patient	Providers/staff
Gender	Male or Female	Male or Female
Age	13 years old and above	-
Experience	Received any types of drug treatment and rehabilitation programmes at the C&C1M Clinics	Involved in the implementation of drug treatment and rehabilitation programmes at the C&C1M Clinic and possess at least one (1) year of work experience in the job scope

The study employed two sampling techniques, namely purposive and snowball sampling technique. Purposive sampling technique is the process of selecting a sample by using a strategic way; whereby the unit of analysis is selected according to specific criteria, determined in a study (Bryman, 2012). The sampling process directly focused on the clients, the C&C1M Clinics, NADA, and MOHA. Snowball sampling technique, on contrary, allows more participants to be recruited in this study. Bryman [24] explains that snowball sampling technique is the process of collecting a sample by initiating the search within a small group of people; these participants would then recommend other participants who are perceived to be relevant for the study. The final sample size of thirty-seven (37) participants was deemed sufficient, as no new themes emerged, indicating that data saturation had been reached.

### The interview guide (validation and reliability)

Semi-structure interviews were adopted in this study, as it fit the qualitative design of the study. A semi structured interview is beneficial when investigating complex and sensitive issues [25]. Such method was selected as it can guide the interview process, by providing standard questions, and create flexible flow of communication during the interaction between the participants and researcher. Bryman [24] mentions that, semi-structured interview is an interview conducted based on interview guide, which comprises a series of general and significant questions, to be asked to the participants.

All the semi-structured questions were developed based on the recommendations by Paton [26]. The six common types of interview questions are (1) experience and behaviour, (2) opinions and values, (3) feeling, (4) knowledge, (5) sensory, and (6) background or demographic profile. These questions represent different time frames namely, the past, present, and future. Therefore, this study employed the guideline recommended by Paton [26] when designing comprehensive, reliable, and valid instruments.

Apart from that, to enhance the validity of the instruments, two experts reviewed the questions. The experts were chosen based on their expertise, capabilities, and experiences particularly in drug treatment and rehabilitation programmes and effectiveness study in Malaysia (see appendix). To ensure the trustworthiness of the findings, this study employed member checking techniques to enhance the credibility and reliability of the results. This strategy involved asking participants (the providers) to verify the accuracy of their experiences by reviewing the study’s final report and confirming that the findings accurately reflected their perspectives and experiences [27].

### Reflexivity and researcher positionality

The research team comprised a number of academics and postgraduate students with considerable experience in qualitative research and drug-related studies in Malaysia. Their academic and research backgrounds provided valuable contextual understanding of the study setting and facilitated rapport building with participants. Prior to the data collection, the participants were informed of the researchers’ academic affiliations and the purpose of the study. No prior personal relationships existed between the researchers and participants. To minimise potential bias, reflexivity was practised throughout the research process. Regular discussions were conducted by the research team to critically examine emerging interpretations and challenging underlying assumptions after in-dept interview sessions involving each group of the participants. These measures helped ensure that the findings remained grounded in the participants’ perspectives, thereby enhancing the credibility and trustworthiness of the study.

### Interview procedure and data collection

Prior to the data collection, the researchers engaged in formal and informal communications with relevant agencies and centre representatives. This approach provided a clear understanding of the right procedures and processes for data collection to both the researchers and responsible agencies. The persons in charge of the agencies set up a schedule to undertake the in-depth interviews with the participants. The participants were given the explanation on the procedures of the interview as stated in the informed consent. This was to ensure that the participants were fully informed about the study’s purpose, procedures, and their right to withdraw at any time without consequence. The participants were also informed regarding the confidentiality and anonymity of the data collected. The researchers were also not allowed to record any pictures of the participants as stipulated in the rules and regulation by the C&C1M, NADA, and MOHA. Then, few minutes were given to the participants to read and sign the informed consent forms provided. The in-depth interviews were conducted in both Malay and English language depending on the participants’ preference, for 20 min to 1 h and the entire sessions were recorded using voice recording tools. The in-depth interviews were conducted by three female researchers, comprising postgraduate students and lecturers with prior training in qualitative research methods, as indicated in the COREQ checklist. All interviewers were involved in the data collection process.

### Data analysis

The data analysis started with the process of transcribing the interviews. According to Ahmad et al. (2017), at least three (3) months are required to prepare for data analysis. Thus, prior to the data analysis, the process of transcribing of the data (from audio to text) collected was undertaken. Thick and rich descriptions of the data were turned into text to ensure the validity of the data collected. In the process of data reduction, narrative analysis was conducted to discover the socio-demographic patterns of the participants. To identify themes, categories, patterns and to compare and confirm the data, this study also adopted thematic analysis and cross case synthesis.

Thematic analysis was conducted based on Braun and Clarke’s [28] guideline. Firstly, the researchers familiarised themselves with the data. This was done by reading the transcripts of the in-depth interviews multiple times to fully understand the raw data obtained. Initial coding was conducted independently by researchers using NVivo software, which was used to organise, manage, and retrieve coded data systematically. The generated codes were then reviewed and compared to identify any similarities and differences across cases. Next, the researchers carefully modified and systematically arranged the themes based on the research objectives. The themes were analysed, particularly to identify the relationship between the themes and subthemes. At this step, the illustrations of the themes were generated by using NVivo. Three researchers were involved in synthesising the codes and themes of the study.

In doing so, direct quotations were utilised to avoid bias or misinterpretation of the data. To analyse the data, data triangulation process was also conducted upon converging and confirming the data based on the participants’ and key persons’ perspectives, determined through in-depth-interviews. The triangulation process helped ensure the validity of the findings. Bryman [24] defines triangulation as the use of more than one source of data in a study to crosscheck or validate the findings of the study. The preliminary findings also were reviewed by experts from the National Anti-Drugs Agency (NADA) to assess the credibility and relevance of the interpretations.

### Ethics approval

As part of the requirements to ensure the reliability and validity of the research, particularly in relation to the methodology and research instruments, ethical approval was obtained from the Universiti Teknologi MARA Research Ethics Committee (REC) [Ref: REC/403/17 600-IRMI (5/1/6)].

### Findings

#### Profile of participants

The participants in this study comprised the receivers, patients, or clients of drug treatment centres and providers, represented by staff of the centres, key providers from the C&C1M Clinics, NADA, and MOHA (Refer Table 2).

**Table 2:** Profile of service recipients

No.	Item	Category	Frequency (*N*)			
			Clinic 1	Clinic 2	Clinic 3	Clinic 4
1.	Gender	Male	7	7	7	4
		Female	0	0	0	1
2.	Age (years old)	13–18	0	2	0	0
		19–39	6	5	6	4
		40≥	1	0	1	1
3.	Marital status	Single	5	5	0	1
		Married	2	2	4	3
		Divorced	0	0	3	1
4.	Education level	Secondary school	0	0	0	1
		SPM/SPMV	5	0	1	1
		Diploma	0	2	1	1
		Degree	0	0	1	0
		Schools dropout	0	4	0	0
		Not declared	2	1	4	3
5.	Total number of participants		7	7	7	5

A total of 26 participants participated in this study. Overall, the majority of the participants were male and all participants represented diverse backgrounds in terms of age, marital status, and education background. In addition, 11 key persons representing service providers were involved in the study. All the key persons involved had extensive experiences in the treatment and rehabilitation of drug use disorder. The opinions and insights shared in the in-depth interviews were reflective of the points of view held by their respective agencies.

### Resilience of the participants

The resilience of the participants was developed from the treatment and rehabilitation programmes offered by the C&C1M clinics. The key factors of resilience discovered consisted of (1) the guidance, support, and trust that clients received during their recovery and (2) post-treatment and rehabilitation programme enhancement (Refer Table 3).

**Table 3:** Resilience of the participants

Theme	Sub theme
Key factor of resilience	1a. Guidance, support, and trust received during recovery
	1b. Post-treatment and rehabilitation programme enhancement
Barrier to resilience	2a. Clients’ self-reliance
	2b. Limited Institutional support

### Theme: Key factors of resilience development

#### 1a. Guidance, support, and trust received during recovery

The findings show that drug treatment and rehabilitation programmes play a meaningful role in addressing drug use disorder by providing a structured and controlled environment for recovery. Within this setting, participants appeared to gradually develop resilience through consistent guidance, emotional support, and a sense of trust in the rehabilitation process.

This is my second time receiving treatment, and the staff have given me a lot of support […] sometimes they give me opportunities to speak and even take on a coordinator role in the centre […] my responsibility is greater because I have to take care of other clients. (Participant, Male, Clinic 1).

The environment of the centre was perceived as supportive and conducive to recovery. Participants were able to engage in daily activities that were not only structured but also purposeful, allowing them to rebuild routines and remain in recovery. The participants from Clinic 1 widely perceived the programmes to be effective; however, they acknowledged that success ultimately depends on their own ability to manage triggers and maintain self-control.

What stood out was the role of staff in shaping this recovery experience. Participants highlighted the importance of continuous guidance, which goes beyond instruction and extends to emotional reassurance and trust-building. Participants’ confidence in the staff’s professional background further strengthens their willingness to engage with the programmes. At the same time, exposure to relevant knowledge and rehabilitation techniques helped participants better understand their condition and the steps needed to avoid relapse.

Here, I have been exposed to some knowledge […] I have learned about rehabilitation practices, and this has influenced me to feel that I no longer want to use drugs. (Participant, male, Clinic 4).

Taken together, these findings suggest that resilience is not developed in isolation, but rather through a supportive environment where trust, guidance, and structured engagement are consistently present. In this context, the effectiveness of the programmes particularly in the early stages of recovery appears closely linked to the stability and support provided within the centre. This underscores the importance of strengthening structured psychosocial programmes within rehabilitation centres as a foundation for building sustained resilience during early recovery.

#### 1b. Post-treatment and rehabilitation programme enhancement

While the programmes demonstrate clear benefits during the treatment phase, their sustainability beyond the centres remains uncertain. Participants described facing significant challenges once they returned to their daily environments. This includes exposure to peers who continue to use drugs, easy access to substances, and experiences of “*rindu*” an intense craving that is difficult to manage.

Yes, it is quite effective […] before this, my addiction behaviour was very bad […] I was a heavy drug user. (Male, Clinic 3).

I think I will not abuse drugs anymore, I have already become fed up, and my age already 55 years old. (Male, Clinic 1)

Relapse is often associated with familiar environments, emotional distress, and ongoing life pressures. Participants also pointed to specific situations such as returning to places linked to previous drug use as triggers that can quickly undermine recovery efforts. These realities highlight a gap between structured rehabilitation settings and the complexity of real-life environments.

Another concern raised was the limited availability of follow-up support after discharge. Without consistent monitoring or continued engagement, participants may struggle to maintain the progress achieved during treatment. This scenario is partly shaped by institutional constraints, which restrict the extent of post-treatment intervention.

C&C only provide drug treatment and rehabilitation to the client […] when the client returned to their hometown no more treatment from C&C, so it is difficult for us to make sure the client still recover, in this centre they are our responsibility. (Key Person, male, Clinic 1).

As a result, participants expressed a clear preference for extended or continuous rehabilitation support. Though their reliance on the centres reflects the effectiveness of the programmes, there is a noticeable lack of external support systems that can sustain the participants’ recovery. This suggests that resilience, while developed during treatment, requires ongoing reinforcement through post-treatment interventions to remain effective over time. This highlights a gap in the continuity of care after discharge and underscores the need for strengthening the support mechanisms of post-treatment and rehabilitation programmes in the policy and implementation of drug treatment and rehabilitation programmes in Malaysia.

### Theme: Barriers to resilience

#### 2a. Clients’ self-reliance

The findings also point to structural and operational limitations that affect programme implementation at the C&C1M clinics. In many instances, the programmes appeared to function in a reactive manner, largely due to constraints in facilities, staffing, and capacity. These limitations restrict the full delivery of planned modules and reduce the clinics’ ability to respond to the increasing number of clients requiring treatment.

From the key person’s perspective (Clinic 4), although multiple rehabilitation modules were in place, there was no certainty that clients would sustain recovery after leaving the centre. This uncertainty reflects the broader challenge of translating short-term intervention into long-term behavioural change.

C&C1M Clinics only provides treatment and rehabilitation while the clients are in the centre […] once they return to their hometown, there is no further treatment from C&C1M, so it is difficult for us to ensure that they remain in recovery […] here in the centre, they are our responsibility. (Key Person, Male, Clinic 1).

#### 2b. Limited institutional support

Some participants expressed the desire to remain within the programmes for a longer period, indicating a continued dependence on structured support. However, the clinics face difficulties in monitoring the participants after discharge. Follow-up efforts such as phone calls, social media communications, or visits are not systematically implemented and often depend on individual staff’s initiatives.

For me, yes, I want to leave this centre, but I am not strong […] I am not strong […] that is why if I break my connection with this centre, I will most likely relapse. (Participant, Male, Clinic 1).

This raises important questions about responsibility in the recovery process. While service providers play a critical role during treatment, sustained recovery cannot rely solely on institutional support. Instead, it requires a broader network of responsibility, including community involvement and, most importantly, the individuals’ own capacity to manage their condition.

In this regard, self-reliance emerges as a crucial component of long-term resilience. Participants must gradually transition from dependence on structured programmes to developing the internal capability to regulate behaviours, cope with triggers, and sustain recovery independently.

## Discussion

The findings suggest that resilience in drug recovery is closely linked to the structured and supportive environment provided by rehabilitation centres. In this study, participants clearly associated their progress with the presence of guidance, emotional support, and trust from staff. This indicates that, particularly in the early stages, resilience is not entirely self-driven but is shaped through consistent external support. This is consistent with the international standards for the treatment of drug use disorders, which emphasise that recovery activities should enhance patient resilience and support social reintegration for sustained recovery [3].

However, this form of resilience appears to be situational. Once participants return to their everyday environments, many of them face difficulties in sustaining recovery. Exposure to previous social circles, accessibility of drugs, and emotional pressures were repeatedly mentioned as factors that weaken their ability to remain drug-free. This may be explained by the need for a stable and supportive environment in the development of resilience [17]. This suggests that the transition from a controlled environment to an unstructured setting remains a critical point of vulnerability, which requires support from the centres, families, or other relevant stakeholders to sustain recovery.

Another issue that emerges from the findings is the limited continuity of support after discharge. Both clients and providers acknowledged that monitoring mechanisms are not consistently implemented once individuals leave the centres. In this context, recovery appears to be treated as a completed phase rather than an ongoing process. As reflected in several clinics, participants continue to rely on institutional support, indicating that resilience has not yet translated into independent coping capacity.

At the same time, structural limitations within the rehabilitation system also shape these outcomes. Constraints related to staffing, facilities, and monitoring capacity affect how programmes are delivered and sustained over time. This issue is consistent with previous studies that highlight limitations among service providers [29, 30]. These challenges suggest that the issue is not solely individual, but also embedded within institutional and systemic conditions.

Importantly, the findings point to an incomplete transition from dependence to self-reliance. While participants demonstrate positive changes during treatment, many still express uncertainty about managing recovery on their own. This indicates that self-reliance should not be assumed as an automatic outcome of rehabilitation, but rather as a gradual process that requires both internal development and continued external reinforcement [31]. Thus, sustaining recovery requires more than short-term intervention. It involves a continuous interaction between individual capability, institutional support, and the broader social environment, highlighting the need for a more integrated and sustained recovery approach.

Collectively, resilience is a dynamic process that can protect individuals from engaging in drug-related activities. Coping strategies at the individual, family, and community levels may contribute to the development of resilience. This finding is consistent with that of Calpe-Lopez et al. [13], who highlight the role of coping mechanisms in fostering resilience among individuals with drug use disorder. The recovery process largely depends on the individual’s own commitment and efforts, while social and economic factors serve as important supporting influence.

### Recommendations

From the findings and discussion of this paper, three recommendations to enhance the resilience of individuals involved with drug use disorder are proposed as follows (1) emphasising aftercare services; (2) empowering clients; and (3) strengthening policy implementation, monitoring, and evaluation. It is crucial that all efforts and strategies involve the participation and support of all stakeholders.

#### Emphasising “Aftercare services”

This study highlights the importance of aftercare services in supporting participants/clients to sustain their recovery beyond the treatment phase. The findings indicate that while dug treatment and rehabilitation programmes implemented at the C&C1M clinics are beneficial, their effectiveness is largely confined to the duration of the programmes. Long-term recovery remains uncertain due to various relapse triggers encountered after discharge [32].

Currently, participants are only required to submit monthly reports to the district police stations upon completing treatment. Maintaining a continuous connection between clients and the C&C1M clinics could further strengthen recovery outcomes [33]. Such continuity may provide ongoing guidance and emotional support, which are critical in preventing relapse. Nevertheless, extending aftercare services may increase the workload of rehabilitation centres, particularly in sustaining long-term engagement with former clients. 

To address this, supportive interventions should be tailored according to the specific needs and characteristics of different client groups. A segmented and targeted approach would enable more effective intervention strategies in addressing issues related to drug use disorder [34].

In addition, the scope of monitoring could be expanded by involving key community stakeholders, including family members, local communities, village heads, and residential associations [35]. These actors can play a supportive role in overseeing clients’ recovery and facilitating their reintegration into society. However, such monitoring efforts should remain under the supervision of relevant authorities, particularly the National Anti-Drug Agency (NADA) and the C&C1M clinics, to ensure consistency and accountability.

At the same time, there is a need to strengthen public awareness regarding the shared responsibility in addressing drug use disorder, especially within immediate social environments. Equipping communities with basic knowledge and skills can enhance their capacity to support former drug users during reintegration.

Nevertheless, societal involvement remains complex. The lack of open communication and persistent stigma towards individuals with drug use disoder continue to hinder effective community support. This is further compounded by competing priorities, such as employment commitments, security concerns, and limited awareness of the communities role in addressing drug-related issues. While some members of society may not be indifferent, their limited engagement may stem from the perception that drug rehabilitation is beyond their professional or personal responsibility.

#### Empowering clients

This study highlights the importance of empowering clients as a strategy to strengthen their capacity to prevent relapse and manage relapse triggers. The findings indicate that clients who undergo drug treatment and rehabilitation programmes are capable of experiencing meaningful personal transformation and growth. Building on this, the NADA and rehabilitation centres can further support recovery by providing opportunities for clients to actively contribute to the rehabilitation environment.

One potential approach is the implementation of a ‘buddy system’, where clients take on supportive roles such as mentoring newly admitted individuals. This approach allows clients to develop a sense of ownership over their recovery while simultaneously assisting others in managing relapse triggers. In doing so, the buddy system fosters confidence, trust, and accountability, while also providing positive role models within the rehabilitation setting. It may also function as a preventive mechanism against negative experiences, such as social isolation, bullying, or exposure to relapse triggers [36–38].

In addition, clients who have been discharged from the C&C1M clinics could be engaged as ambassadors for the centres. These individuals can play a valuable role in promoting the rehabilitation programmes by sharing their experiences with peers and other individuals seeking support. Through the dissemination of knowledge, personal recovery journeys, and lessons learned from past relapse experiences, former clients can provide relatable and credible sources of encouragement for others facing similar challenges.

Engagement in peer mentoring and ambassadorial roles may therefore contribute to strengthening both individual and collective recovery processes. However, the success of such initiatives depends on the active participation and collaboration between clients and service providers. When effectively implemented, these strategies have the potential to enhance programme effectiveness by fostering a supportive environment, strengthening internal resilience, and promoting sustained recovery through peer-driven empowerment [39].

#### Strengthening policy implementation, monitoring, and evaluation

The role of policy evaluation must be strengthened at all levels of service providers and recipients, specifically including MOHA, NADA, C&C1M clinics and their clients, and other stakeholders such as families, communities, and local governments. To do this, the ministry should strengthen its monitoring and evaluation role by adopting a participatory monitoring and evaluation approach [40]. The strategy implemented by Vernooy et al. [41] has been shown to enhance the effectiveness of programmes and projects. This strategy requires the monitoring and evaluation process to not solely depend on MOHA, NADA, and C&C1M clinics, but also other essential stakeholders such as families, communities, employees, and others.

This strategy encourages collaboration among various stakeholders to collectively achieve success and gain knowledge from one another. It also empowers the local community to actively participate and fulfil their responsibilities in the implementation of drug treatment and rehabilitation programmes [42]. Consequently, the monitoring and evaluation process would not solely rely on NADA. While NADA is the primary agency responsible for addressing drug use disorder in the country, and is supported by other agencies such as the Royal Malaysia Police and the Immigration Department, MOHA should consistently strengthen its overall role from top to bottom after endorsing new policies, guidelines, or regulations pertaining to drug issues, specifically in relation to drug treatment and rehabilitation programmes [43].

An additional factor to consider is enhancing the role of NADA and MOHA in policy assessment through the implementation of participatory monitoring and evaluation systems as the standard approach. Although there are existing systems in place, it is crucial to prioritise appropriate evaluation criteria. Without suitable key criteria to assess programme effectiveness, ministries, agencies, and other stakeholders may only address the symptoms of the problem rather than its root causes. Consequently, this may lead to inefficient use of financial resources, labour, and time in government-led evaluation programmes.

The institution should establish a suitable set of criteria by obtaining input from actual implementers of the drug treatment and rehabilitation programmes, such as counsellors, case officers, and other relevant personnel at the grassroots level. Such input can be gathered through research and innovation activities conducted or organised by NADA or C&C1M clinics.

Although there may be changes in leadership, the drug treatment and rehabilitation programme blueprint should incorporate policy evaluation initiatives. This ensures that any changes in leadership do not disrupt the evaluation process, but instead support continuous improvement in the implementation of evaluation approaches.

At the same time, the continuous development of communication technologies provides the opportunities to strengthen monitoring and evaluation processes. Therefore, continuous monitoring and evaluation not only maintain or enhance the effectiveness of drug treatment and rehabilitation programmes, but also promote learning and improve stakeholders’ understanding [44, 45]. Overall, this will benefit both the clients and providers of drug treatment and rehabilitation programmes.

### Limitations of study

The insights presented in this study are limited to C&C1M Clinics’ drug treatment and rehabilitation centres. Future research could expand the scope by including other types of treatment and rehabilitation programmes to further explore the importance of developing resilience among individuals with drug use disorder.

Future studies may also benefit from employing different qualitative approaches, such as focus group discussions and observations, to gain a deeper understanding of the issue. Such efforts would contribute to the development of more comprehensive strategies and solutions to address drug abuse, drug use disorder, and related challenges.

While this study was grounded in the Malaysian context, the findings may be relevant to similar rehabilitation settings internationally, highlighting the universal importance of supportive environments, coping resources, and resilience-building processes in sustaining recovery. As drug use disorder remains a global concern, these insights may inform resilience-oriented approaches to treatment and rehabilitation across different national contexts.

## Conclusion

The treatment and rehabilitation approaches of the centres may be effective within their institutional settings but remain insufficient in preparing individuals for sustained recovery in real-world environments. The experiences and insights gained from this study can significantly contribute to the development of more comprehensive and effective treatment and rehabilitation programmes for future generations. Resilience is a protective factor that supports sustained recovery among individuals with drug use disorder. A strong and positive network of numerous stakeholders, including individuals, families, society, communities, the private and public sectors, and non-governmental organisations, is also needed to break the complex drug abuse cycle. Strenghtening resilience through coordinated efforts among individuals, families, communities and institutions is essential for promoting sustained recovery and reducing the long-term impact of drug use disorder.

## Supplementary Information

Below is the link to the electronic supplementary material.

Supplementary Material 1

Supplementary Material 2

## Data Availability

No datasets were generated or analysed during the current study.

## Abbreviations

C&C1M Clinics: Cure & Care 1Malaysia Clinics
MOHA: Ministry of Home Affairs, Malaysia
NADA: National Anti-Drugs Agency
PUSPEN: Narcotics and Drug Treatment Centres
REC: Research Ethics Committee
SPM/SPMV: Malaysian Certificate of Education
