# Supplementary material for: Resilience in drug recovery: insights from treatment and rehabilitation programmes in Malaysia
Source: Subst Abuse Treat Prev Policy. 2026 Jul 15;21:67. doi: 10.1186/s13011-026-00745-2 (PMC13670526; doi:10.1186/s13011-026-00745-2)
Supplement: Supplementary file 1 [file 13011_2026_745_MOESM1_ESM.docx]

**Appendix: List of Semi-Structure Interview Questions**

| **Section** | **Questions** |
| --- | --- |
| Introduction | **Ice-breaking Session**  *(to build rapport between the participant and the interviewer)* |
| A  Background/ Demographic | 1. What is your age? 2. What is your current health status?  *(Probe further regarding relapse status or the presence of any health conditions.)* 3. Do you have any family members who are still involved in your life? 4. What type of treatment and rehabilitation programme have you received? |
| B  Experience and Behaviour/Opinion and Values/Feeling/ Knowledge and Sensory | 5. How long have you been receiving treatment and rehabilitation at this centre? 6. Who encouraged you to come to this centre? 7. Are you happy being in this centre? *(Probe further regarding participants’ perspectives on: (a) treatment and rehabilitation activities; and (b) the centre environment.)* 8. Would you mind sharing the type of drug(s) you have used? *(Probe further regarding the chronology of the participant’s drug use disorder.)* 9. What factors contributed to your involvement in drug use disorder? *(Probe further regarding factors that support drug recovery and resilience, as well as barriers encountered in maintaining a drug-free life.)* 10. How have you achieved recovery or ceased drug use? |
