# Supplementary material for: Resilience in drug recovery: insights from treatment and rehabilitation programmes in Malaysia
Source: Subst Abuse Treat Prev Policy. 2026 Jul 15;21:67. doi: 10.1186/s13011-026-00745-2 (PMC13670526; doi:10.1186/s13011-026-00745-2)
Supplement: Supplementary file 2 [file 13011_2026_745_MOESM2_ESM.docx]

**Appendix:

RESILIENCE IN DRUG RECOVERY: INSIGHTS FROM TREATMENT AND REHABILITATION PROGRAMMES IN** **MALAYSIA**

**COnsolidated criteria for REporting Qualitative studies (COREQ) Checklist (Qualitative Research Reporting)**

| No | Item | Guide questions/description | Description | Reference page |
| --- | --- | --- | --- | --- |
| 1 | Interviewer/facilitator | Which author/s conducted the interview? | Three authors involved in data collection | 23 |
| 2 | Credentials | What were the researcher’s credentials? | The researchers have demonstrated expertise in qualitative research, with experience in designing and conducting studies involving interviews with vulnerable groups, and analysing data using systematic thematic approaches. *(The researchers' credentials and professional backgrounds were documented in the ethics application and approved by the university's Research Ethics Committee.)* | 9, 22 |
| 3 | Occupation | What was their occupation at the time of the study? | Postgraduate students and lecturers *(The researchers' professional backgrounds and roles were disclosed to participants and were acknowledged as part of the ethical approval process granted by the university's Research Ethics Committee.)* | 9 |
| 4 | Gender | Was the researcher male or female? | Female | Not relevant for this study |
| 5 | Experience and training | What experience or training did the researcher have? | The researchers had prior experience in conducting qualitative research, including in-depth interviews and thematic analysis. | 9 |
| 6 | Relationship established | Was a relationship established prior to study commencement? | Rapport was established between the researchers and participants before and during the interview process. | 8, 9 |
| 7 | Participant knowledge | What did participants know about the researcher? | A briefing was conducted before interviews to inform participants about the study | 9, 10 |
| 8 | Interviewer characteristics | What characteristics were reported about interviewer? | The interviewers were trained in qualitative research methods and had prior experience conducting qualitative interviews. The research team also had experience in drug treatment and rehabilitation research. | 9 |
| 9 | Methodological orientation | What methodology underpins the study? | Qualitative, case study | 5 |
| 10 | Sampling | How were participants selected? | Participants were selected by the center and were required to have the ability to respond to the interview questions | 7 |
| 11 | Method of approach | How were participants approached? | Participants were approached following approval and standard procedures set by the National Anti-Drug Agency and the Cure & Care Centre | 7, 10 |
| 12 | Sample size | How many participants were in the study? | 37 participants | 7 |
| 13 | Non-participation | How many refused or dropped out? | None | - |
| 14 | Setting | Where was data collected? | Data were collected at the drug treatment and rehabilitation centre and related agency with necessary approvals | 7 |
| 15 | Presence of others | Was anyone else present? | No | 8 |
| 16 | Description of sample | Characteristics of sample? | Male and female participants who had experience in drug treatment and rehabilitation programmes | 11 - 12 |
| 17 | Interview guide | Were guides provided/pilot tested? | The study referred to interview protocol endorsed by the Research Ethics Committee of Universiti Technologi MARA (UiTM) | 11 and appendix (interview protocol) |
| 18 | Repeat interviews | Were repeat interviews carried out? | No | - |
| 19 | Recording | Audio/visual recording used? | Yes, audio | 10 |
| 20 | Field notes | Were field notes made? | No | - |
| 21 | Duration | Duration of interviews? | 20 minutes to 1 hour | 10 |
| 22 | Data saturation | Was data saturation discussed? | Yes | 8 |
| 23 | Transcripts returned | Were transcripts returned? | No | - |
| 24 | Number of coders | How many coded data? | - | - |
| 25 | Coding tree | Was coding tree described? | Yes | 12 |
| 26 | Themes derivation | How were themes derived? | Thematic analysis | 10 |
| 27 | Software | What software used? | NVivo | 10 |
| 28 | Participant checking | Did participants check findings? | Yes | 11 |
| 29 | Quotations | Were quotations presented? | Yes | 12, 13, 14, 15 |
| 30 | Consistency | Data consistent with findings? | Yes | 12 |
| 31 | Major themes | Were major themes clear? | Yes | 12 |
| 32 | Minor/sub themes | Were minor/sub themes described? | Yes | 12 |

*Adapted from Stein, R. C., Dunn, J. A., Bartlett, J. M., Campbell, A. F., Marshall, A., Hall, P., Rooshenas, L., Morgan, A., Poole, C., Pinder, S. E., Cameron, D. A., Stallard, N., Donovan, J. L., McCabe, C., Hughes-Davies, L., Makris, A., & OPTIMA Trial Management Group (2016). OPTIMA prelim: a randomised feasibility study of personalised care in the treatment of women with early breast cancer. Health technology assessment (Winchester, England), 20(10), xxiii–201. https://doi.org/10.3310/hta20100*
